# Inter-Skeleton Conductive Routes Tuning Multifunctional Conductive Foam for Electromagnetic Interference Shielding, Sensing and Thermal Management

**DOI:** 10.1007/s40820-024-01540-z

**Published:** 2024-10-28

**Authors:** Xufeng Li, Chunyan Chen, Zhenyang Li, Peng Yi, Haihan Zou, Gao Deng, Ming Fang, Junzhe He, Xin Sun, Ronghai Yu, Jianglan Shui, Caofeng Pan, Xiaofang Liu

**Affiliations:** 1 School of Materials Science and Engineering, Beihang University, Beijing, 100191 People’s Republic of China; 2 Institute of Atomic Manufacturing, Beihang University, Beijing, 100191 People’s Republic of China; 3 Tianmushan Laboratory, Xixi Octagon City, Yuhang District, Hangzhou, 310023 People’s Republic of China; 4 Science and Technology On Electromagnetic Scattering Laboratory, Beijing Institute of Environmental Features, Beijing, 100854 People’s Republic of China

**Keywords:** Inter-skeleton conductive films, Conductive polymer foam, Liquid metal, Electromagnetic interference shielding

## Abstract

Conductive polymer foam (CPF) with excellent compressibility and variable resistance has promising applications in electromagnetic interference (EMI) shielding and other integrated functions for wearable electronics. However, its insufficient change amplitude of resistance with compressive strain generally leads to a degradation of shielding performance during deformation. Here, an innovative loading strategy of conductive materials on polymer foam is proposed to significantly increase the contact probability and contact area of conductive components under compression. Unique inter-skeleton conductive films are constructed by loading alginate-decorated magnetic liquid metal on the polymethacrylate films hanged between the foam skeleton (denoted as AMLM-PM foam). Traditional point contact between conductive skeletons under compression is upgraded to planar contact between conductive films. Therefore, the resistance change of AMLM-PM reaches four orders of magnitude under compression. Moreover, the inter-skeleton conductive films can improve the mechanical strength of foam, prevent the leakage of liquid metal and increase the scattering area of EM wave. AMLM-PM foam has strain-adaptive EMI shielding performance and shows compression-enhanced shielding effectiveness, solving the problem of traditional CPFs upon compression. The upgrade of resistance response also enables foam to achieve sensitive pressure sensing over a wide pressure range and compression-regulated Joule heating function.

**Supplementary Information:**

The online version contains supplementary material available at 10.1007/s40820-024-01540-z.

## Highlights

- Unique inter-skeleton conductive films are constructed in polymer foam.
- The resistance change of the foam can reach four orders of magnitude under compression.
- This foam exhibits strain-adaptive electromagnetic interference shielding performance, anti-interference pressure sensor with high sensitivity over a wide pressure range and compression-regulated Joule heating function.

**Supplementary Information:**

The online version contains supplementary material available at 10.1007/s40820-024-01540-z.

## Graphical abstract

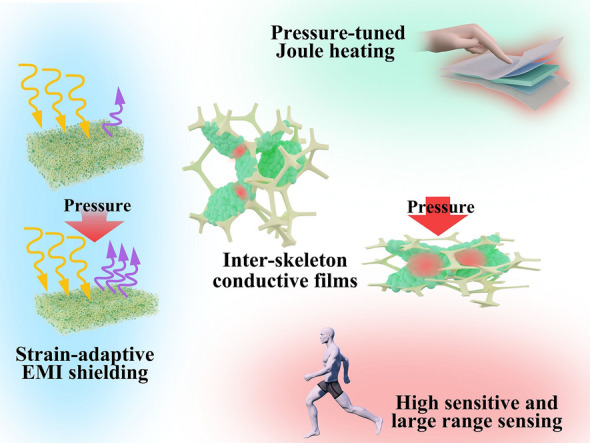

## Introduction

Strain-responsive conductive foam, capable of changing resistance via deformation, plays an essential role in the intelligence development of electronic devices [1–4]. It can integrate multiple functions such as electromagnetic interference (EMI) shielding, pressure sensing, thermal management, and so on, and therefore has been widely used in wearable electronic devices [5–10]. Currently, there is a growing interest in preparing polymer-based conductive foams by coating conductive nanomaterials on the skeleton of polymer foams such as polyurethane foam, polyimide foam, melamine foam, due to their advantages of lightweight, high elasticity, large deformation, good durability, simple fabrication, and low cost [11–13]. However, this type of conductive polymer foam (CPF) still faces an urgent need to improve the resistance response ability to external pressure/strain, which is necessary for function improvement and/or solving application problems.

For example, in the field of EMI shielding that aims at reducing/eliminating the threaten of EM wave pollution to human health and device operation, CPF emerges as a good candidate for the new-generation smart shielding materials with strain-adaptive shielding ability [14, 15]. In principle, the shielding ability of a material is positively correlated with its conductivity and thickness [16–18]. So far, many groups have tried to coat various conductive materials on the surface of polymer skeleton to construct CPFs with compression-tunable conductivity. However, the obtained CPFs generally show limited increase in conductivity upon compression, which cannot offset the negative effect brought by the reduction of foam thickness. As a result, the EMI shielding performances of these CPFs generally decrease as the compressive strain increases, which put wearable devices at risk of EM protection failure during deformation [19, 20]. Moreover, the limited increase in conductivity of CPFs impedes the integration of an efficient sensing function. Currently, improving the sensitivity of sensors over a wide pressure range remains a research hotspot.

**Scheme 1:**
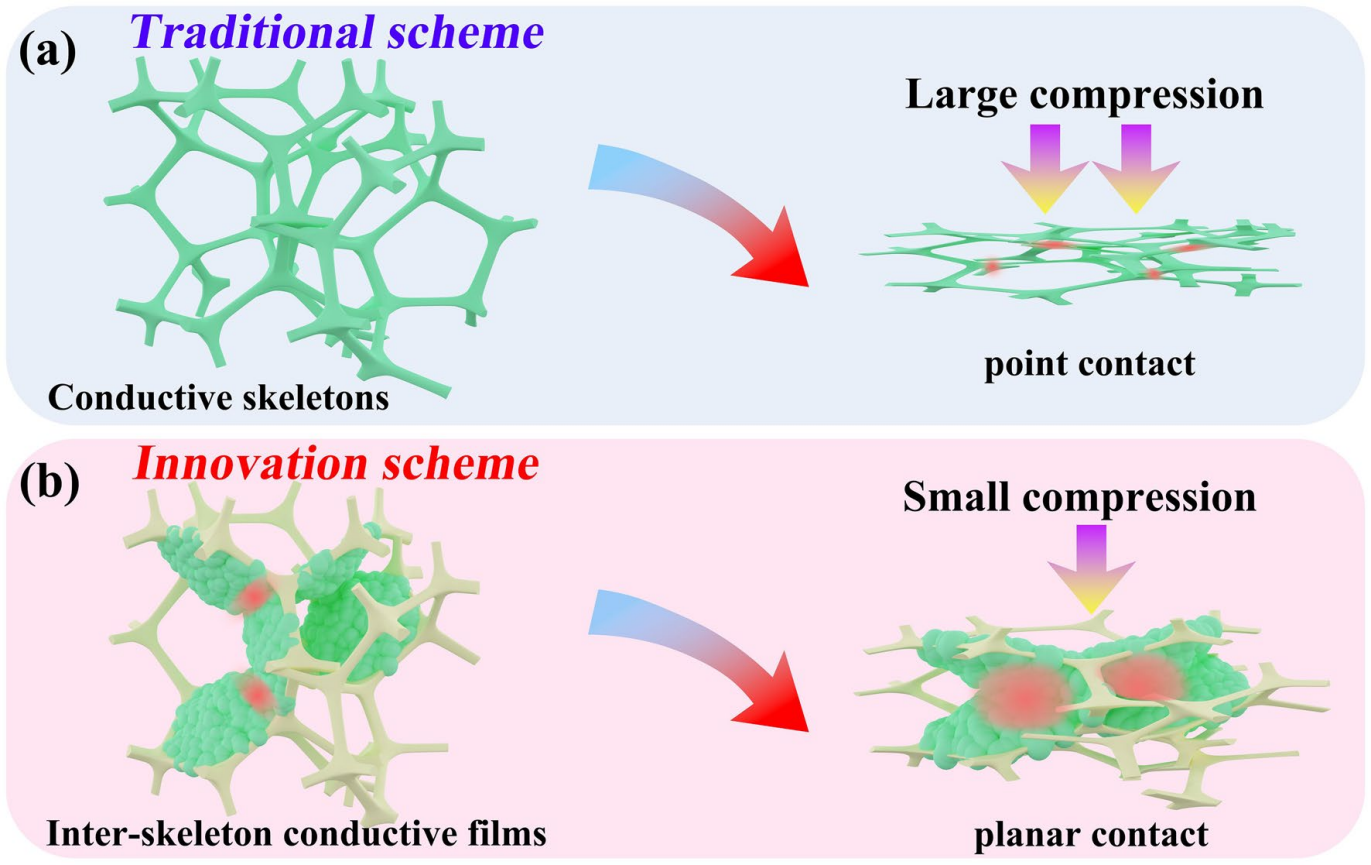
**a** Traditional point contact between conductive skeletons. **b** Innovative planar contact between inter-skeleton conductive films

The resistance change of CPF depends on the formation of temporary contact between conductive components under compression, which increases the conductive paths. The change amplitude of resistance under deformation is affected by multiple factors, such as the number of temporary contacts, contact forms (point/linear/planar contact), pressure/strain [17, 21]. So far, various conductive materials, including 0D nanoparticles, 1D nanowires, and 2D nanosheets, have been explored as coating materials on the foam skeleton (i.e., conductive skeleton) [22, 23]. However, due to the thin thickness of the conductive coating and the large pore size (tens to hundreds of microns) of the polymer foam, the temporary contact of conductive skeleton is too limited to lead to a significant increase in conductivity (as illustrated in Scheme 1a). To overcome this limitation, a more effective contact pathway needs to be designed. In our opinion, this could be achieved by fundamentally changing the loading position of conductive components in the foam.

We envisage “upgrading” the sparse point contact between conductive skeletons to dense planar contact between inter-skeleton conductive films (illustrated in Scheme 1b), to significantly increase the resistance change amplitude of CPF. Liquid metal (LM) is chosen as conductive filler because of its good deformability, which is beneficial for releasing high local pressure to avoid the fracture of skeleton and the detachment of conductive component, thereby ensuring good durability. Meanwhile, as LM droplets deform, a larger contact area can be formed between them, thereby further increasing the conductivity [24–26]. Here, we construct polymethacrylate (PMA) films between the skeletons of melamine foam and load alginate-modified magnetic LM (AMLM) droplets on the PMA films to form inter-skeleton conductive films. The resistance of this foam (called AMLM-PM foam) can decrease by four orders of magnitude under compression, far superior to the traditional design. The inter-skeleton films can also improve the mechanical strength of foam, prevent the leakage of LM during deformation, and increase the reflection/scattering of EM wave in foam. Correspondingly, AMLM-PM foam shows strain-adaptive EMI shielding ability, which solves the problem of performance degradation of traditional CPFs during compression. In addition, the novel design enables foam to integrate multiple smart applications, such as pressure sensing with high sensitivity over a wide pressure range, and compression-regulated Joule heating.

## Experimental Section

### Materials

Liquid metal (LM) EGaln (mass ratio of Ga: 68.5 wt%, In: 21.5 wt%, Sn: 10 wt%) was purchased from Changsha Shengte New Material Co., Ltd. Fe particles (1 µm) were purchased from Hefei AVIC Nano Technology Development Co., Ltd. Sodium alginate (M_W_ 200000) was provided by Aladdin Industrial Co., Ltd. Melamine foam was provided by Xuxian sound insulation and thermal insulation building materials Co., Ltd. Polymethacrylate solution (PMA) was provided by Yihui Co., Ltd.

### Sample Preparation

#### Preparation of Sodium Alginate-Coated Magnetic Liquid Metal

LM was added to dilute hydrochloric acid solution (2 mol L^−1^) and stirred for 5 min; then, Fe particles were added to the above suspension and stirred until Fe particles were completely mixed with LM. Subsequently, magnetic liquid metal (MLM) was obtained after washing with deionized water and ethanol for several times to remove the residual hydrochloric acid. The prepared MLM (100 mg) was added to sodium alginate aqueous solution (0.6 wt%, 10 mL) under vigorously stirring. During this process, the MLM droplets were broken evenly and coated with sodium alginate. Finally, AMLM droplets were separated from the solution.

#### Preparation of PMA-Modified Melamine Foam

PMA solution was diluted by deionized water to increase its fluidity. Melamine foam (15 mm × 30 mm × 5 mm) was immersed into the diluted PMA solution and squeezed several times for absorption (this procedure can be viewed in the Video S1). After drying, the PMA-modified melamine foam was obtained. By changing the concentration of PMA solution in deionized water (from 5, 10, 15, 20 to 25 wt%), foams with different contents of PMA film were obtained (the weight ratio of PMA film in PM foam was 60%, 75%, 85%, 90%, 95%, respectively). The melamine foam modified by PMA diluted solution was named 60PM, 75PM, 85PM, 90PM, and 95PM foam, respectively, and the pure melamine foam was named MA foam.

#### Preparation of AMLM Droplets-Loaded PM Foam

AMLM droplets were dropped on the PM foam and then evenly penetrated into the foam via squeezing. After drying, this process was repeated for several times to adhere more AMLM droplets. A series of AMLM-PM foams with different amount of AMLM droplets were prepared by adjusting the addition amount of AMLM droplets in PM foams.

### Characterization

The morphology, microstructure, and element distribution of the foams were characterized by field emission scanning electron microscope (SEM, JEOL-JSM7500) equipped with energy spectrometer (EDS, OXFORD Xplore 30) and transmission electron microscope (TEM, JEOL-JEM2100F). X-ray diffraction (XRD) patterns were obtained using X-ray diffractometer (XRD, RIGAKU d/max-2500) with Cu Kα radiation. Fourier transform infrared spectroscopy (FTIR) in the range of 400–4000 cm^−1^ was recorded on Nicolet 6700 spectrometer. Surface composition of foams was tested by X-ray photoelectron spectroscopy (XPS, Thermo Scientific k-alpha from the USA). The magnetic hysteresis loops for the samples were examined by a Vibrating Sample Magnetometer (LakeShore 7404). The resistance of foams was measured by the two-point probe method. Copper foils were used as electrodes to connect to the opposite sides of the foam. Conductivity (*σ*) was calculated as *σ* = *d*/(*R* × *A*), where *d*, *R*, and *A* are the thickness, resistance, and area of foam, respectively. Contact angles were recorded using an SDC-100 goniometer. The mechanical properties of the foam were tested by Instron 5565, 5KN universal testing machine at a compression speed of 0.25 mm s^−1^. The surface temperature of foam was recorded with an infrared thermometer (TEMPLAB-RS232), and the heat distribution was monitored with infrared thermal imager. The electrochemical workstation was used to record the current signal when the external pressure was applied on the foam in real time. The electromagnetic interference shielding performance was tested on a vector network analyzer (N5234B PNA-L, KEYSIGHT) using a waveguide method. Firstly, the sample was cut into dimensions of 22.86 mm × 10.16 mm × 5 mm. Subsequently, the sample was compressed to the target strain and wrapped with tape to fix the deformation. Finally, the compressed sample was placed into the waveguide holder for measurement. The obtained parameters of *S*_11_, *S*_21_, *S*_12_, and *S*_22_ were used to calculate *A*, *R*, *T*, SE_T_, SE_R_, and SE_A_ according to the following formula:

1 $$ R = \left| {S_{11} } \right|^{2} $$

2 $$ T = \left| {S_{21} } \right|^{2} $$

3 $$ A = 1 - R - T $$

4 $$ {\text{SE}}_{{\text{T}}} = 10\log \left( {\frac{1}{{\left| {S_{21} } \right|^{2} }}} \right) $$

5 $$ {\text{SE}}_{{\text{R}}} = 10\log \left( {\frac{1}{{1 - \left| {S_{11} } \right|^{2} }}} \right) $$

6 $$ {\text{SE}}_{{\text{A}}} = 10\log \left( {\frac{{1 - \left| {S_{11} } \right|^{2} }}{{\left| {S_{21} } \right|^{2} }}} \right) $$

## Results and Discussion

### Preparation and Characterizations of AMLM-PM Foam

**Fig. 1:**
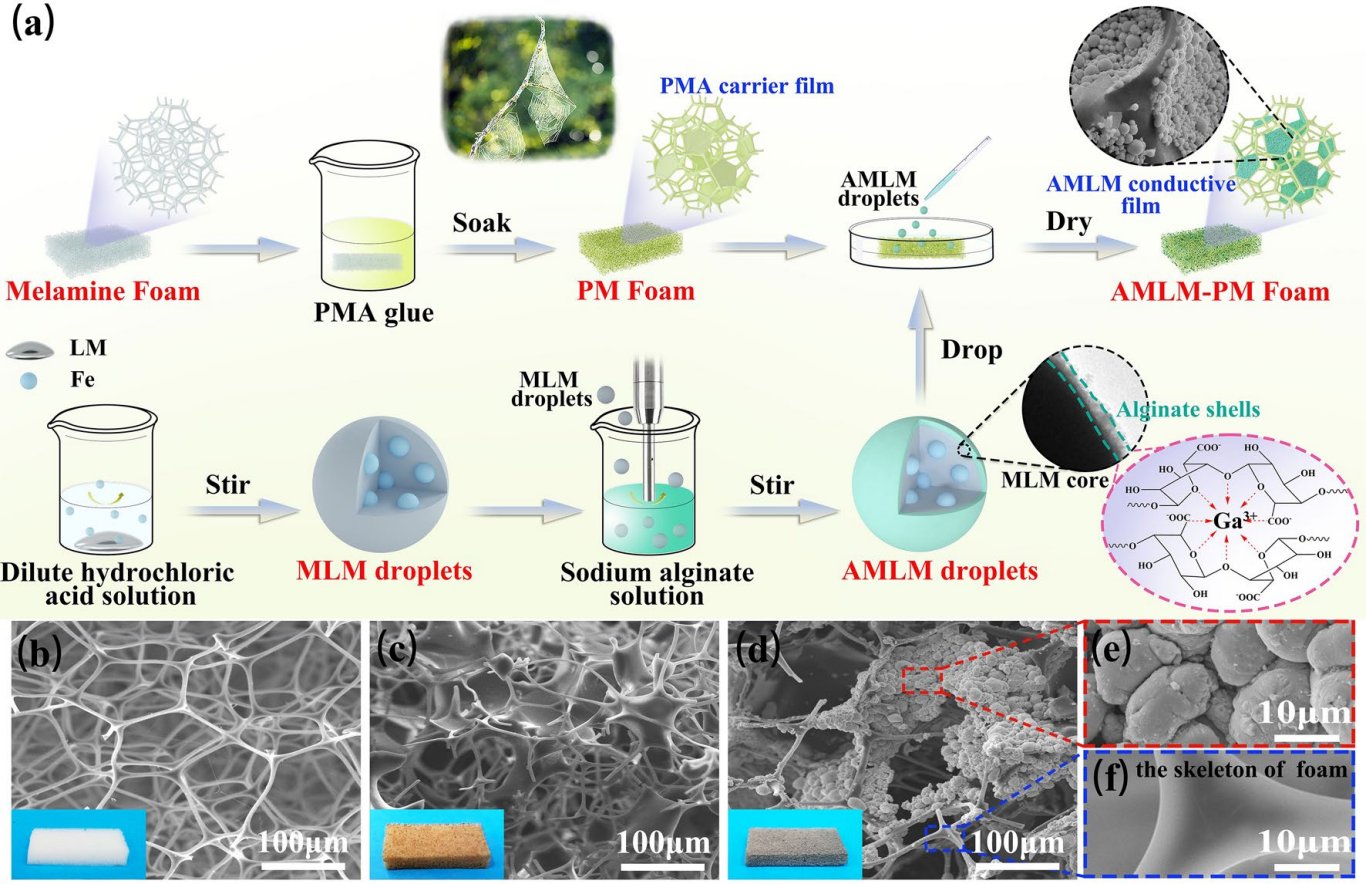
**a** Schematic illustration of preparation of AMLM-PM foam. **b–d** SEM images of melamine foam, PM foam, and AMLM-PM foam, respectively. **e–f** Magnified SEM images of AMLM-PM foam

**Fig. 2:**
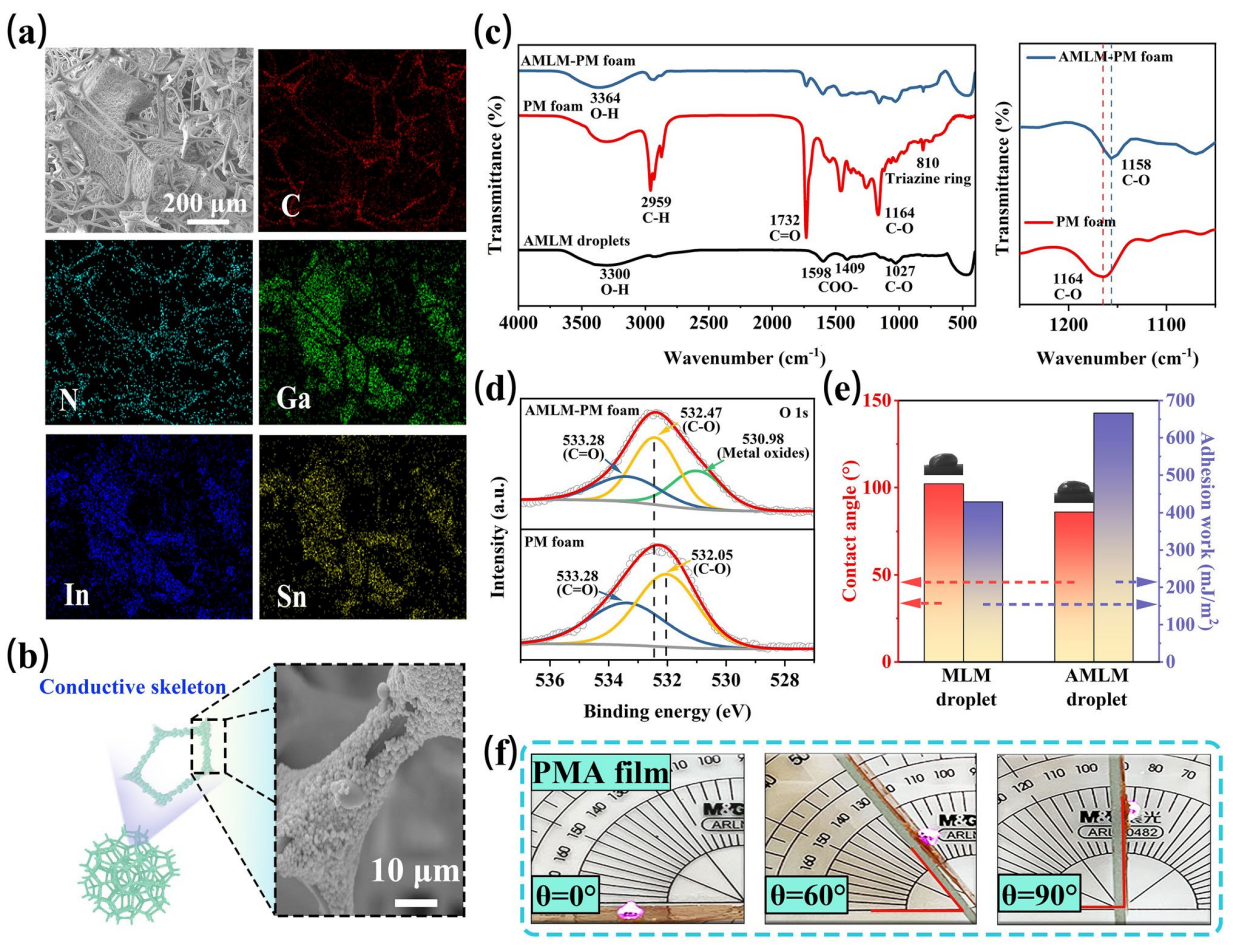
**a** SEM image and EDS mappings of AMLM-PM foam. **b** Scheme and SEM image of AMLM-M foam. **c** FTIR spectra of AMLM droplets, PM foam and AMLM-PM foam, and enlarged image between 1050 and 1250 cm^−1^. **d** High-resolution O 1*s* spectra of PM foam and AMLM-PM foam. **e** Contact angles and work of adhesion (*W*_SL_) of MLM/AMLM droplet on PMA film. **f** Photographs showing the rolling angles of AMLM droplet on PMA film

Figure 1a illustrates the preparation of AMLM-PM foam. Melamine (MA) foam with macroporous structure (pore size of ≈150 µm, Fig. 1b) was used as the elastic skeletons. Polymethacrylate (PMA) was selected to build carrier films between the skeletons, because it has good adhesion to LM by forming hydrogen bonds with gallium oxide on the surface of LM, and is easy to form a film after curing [27, 28]. After multiple immersion in PMA diluted solution, a large number of PMA films are hanged between the melamine skeletons and the macroporous structure of MA foam is well maintained (Figs. 1c and S1a, Video S1). During compression, these PMA films can bend with the deformation of the skeleton, and then return to their original state after releasing the pressure, indicating their flexibility and stability (Fig. S2). The foam turned brown and is denoted as PM foam (i.e., polymethacrylate-decorated melamine foam). To ensure good adhesion between LM and PMA films, we incorporated Fe particles into LM and coated a layer of alginate on the surface of LM droplets, i.e., AMLM droplets (alginate-coated magnetic LM droplets). XRD characterizations and EDS mapping results indicate that Fe particles are physically mixed with LM and uniformly dispersed in LM droplets (Figs. S3 and S4). In this case, the fluidity of LM decreases, which can prevent LM droplets from leaking during foam compression (Fig. S5) [29, 30]. Meanwhile, droplets are endowed with ferromagnetism, which is beneficial for enhancing their response to electromagnetic waves (Fig. S6) [31, 32]. Since the carboxyl groups in the G segment of alginate can chelate with Ga^3+^, a thin layer of gallium alginate gel can uniformly cover the surface of MLM droplets (Figs. S7 and S8) [33–35]. The formation mechanism is explained in the supporting information. Finally, AMLM droplets were dropped on the foam, penetrated into the foam and adhered to the PMA films, forming inter-skeleton conductive films. We carefully optimized the weight ratio of AMLM droplets to PMA films to ensure that AMLM droplets can fully cover the PMA films. Relevant discussion and results can be found in Figs. S9-S11. The optimal weight ratio of AMLM droplets to PMA films is 7/1. The microstructure of AMLM-PM foam and the distribution of AMLM droplets were characterized using SEM and EDS. As shown in Fig. 1d-f and S1b, AMLM droplets are concentrated on the PMA films, while the skeleton of the melamine foam is exposed without AMLM droplets adhesion. The inter-skeleton AMLM conductive films resemble to the spiderweb hanging between tree branches. The EDS mapping results in Fig. 2a show that the signals of C and N elements mainly from the foam skeleton are inconsistent with the signals of Ga, In, and Sn elements. This further proves that AMLM droplets distribute on the PMA film rather than on the foam skeleton, forming AMLM conductive films. Meanwhile, the C signal from the PMA films cannot be detected, which means that the PMA films are completely covered by AMLM droplets. It is worth mentioning that the large size of AMLM droplets ensures that they can selectively adhere on PMA films. In addition, we prepared a control sample by loading small-sized AMLM droplets on the skeleton of melamine foam (denoted as AMLM-M foam, Fig. 2b). The AMLM-M foam has typical conductive skeletons, while the AMLM-PM foam has unique inter-skeleton conductive films.

The chemical interactions of AMLM droplets and PM foam were characterized by FTIR (Fig. 2c). In the spectrum of PM foam, the characteristic peaks at 2959, 1732, and 1164 cm^−1^ correspond to the stretching vibrations of C–H, C=O, and C-O, respectively, and the peak at 810 cm^−1^ derives from the triazine ring of melamine foam. For AMLM droplets, the peaks at 3300 and 1027 cm^−1^ come from the stretching vibrations of O–H and C–O, respectively, and the peaks at 1598 and 1409 cm^−1^ correspond to the stretching vibrations of COO-. In the spectrum of AMLM-PM foam, the intensities of the characteristic peaks from PM foam are weakened, and obvious peak shifts can be detected. For example, the C-O characteristic peak shifted from 1164 cm^−1^ in PM foam to 1158 cm^−1^ in AMLM-PM foam, indicating the change of chemical environment of C-O functional groups after the adhesion of AMLM droplets. Additionally, the O–H characteristic peak in AMLM droplets shows an obvious shift to 3364 cm^−1^ in AMLM-PM foam, which implies the formation of hydrogen bonds between PM foam and the oxygen-containing functional groups on the surface of AMLM droplets. The XPS survey spectrum of AMLM-PM foam indicates the presence of C, O, Ga, In, and Sn elements (Fig. S12). As shown in the high-resolution O 1*s* spectra (Fig. 2d), the peak from C–O group in PM spectrum is located at 532.05 eV, while it shifts to 532.47 eV in AMLM-PM spectrum. This further verifies the formation of hydrogen bond between AMLM droplets and PM foam, which is conducive to enhancing the adhesion of AMLM droplets on PMA films.

Subsequently, we used contact angle testing to indicate the effect of alginate modification on droplet adhesion. As shown in Fig. 2e, the contact angle of unmodified MLM droplet on PMA film is 102.2°. After being coated with alginate, the wettability of AMLM droplet on PMA film is improved, and the contact angle is reduced to 86.1°. The work of adhesion (*W*_SL_) between droplet and film was further calculated according to the Young-Dupre equation (Fig. S13) [28]. The *W*_SL_ value between AMLM droplet and PMA film is 666.4 mN m^−1^, larger than the value between MLM droplet and PMA film (429.1 mN m^−1^) (Fig. 2e). It indicates that the encapsulation of alginate can improve the adhesion between AMLM droplets and PMA carrier films. The obliquity experiment in Fig. 2f further visually demonstrates the good adhesion of AMLM droplet on PMA film. As the inclination angle increased from 0° to 90°, the AMLM droplet did not roll off the PMA film, but remained stationary. The good adhesion of AMLM droplets on PMA films can prevent liquid metals from leaking out of the polymer foam (Fig. S14), which is beneficial for their repeated deformation applications.

### Mechanical and Electrical Properties of AMLM-PM Foam

**Fig. 3:**
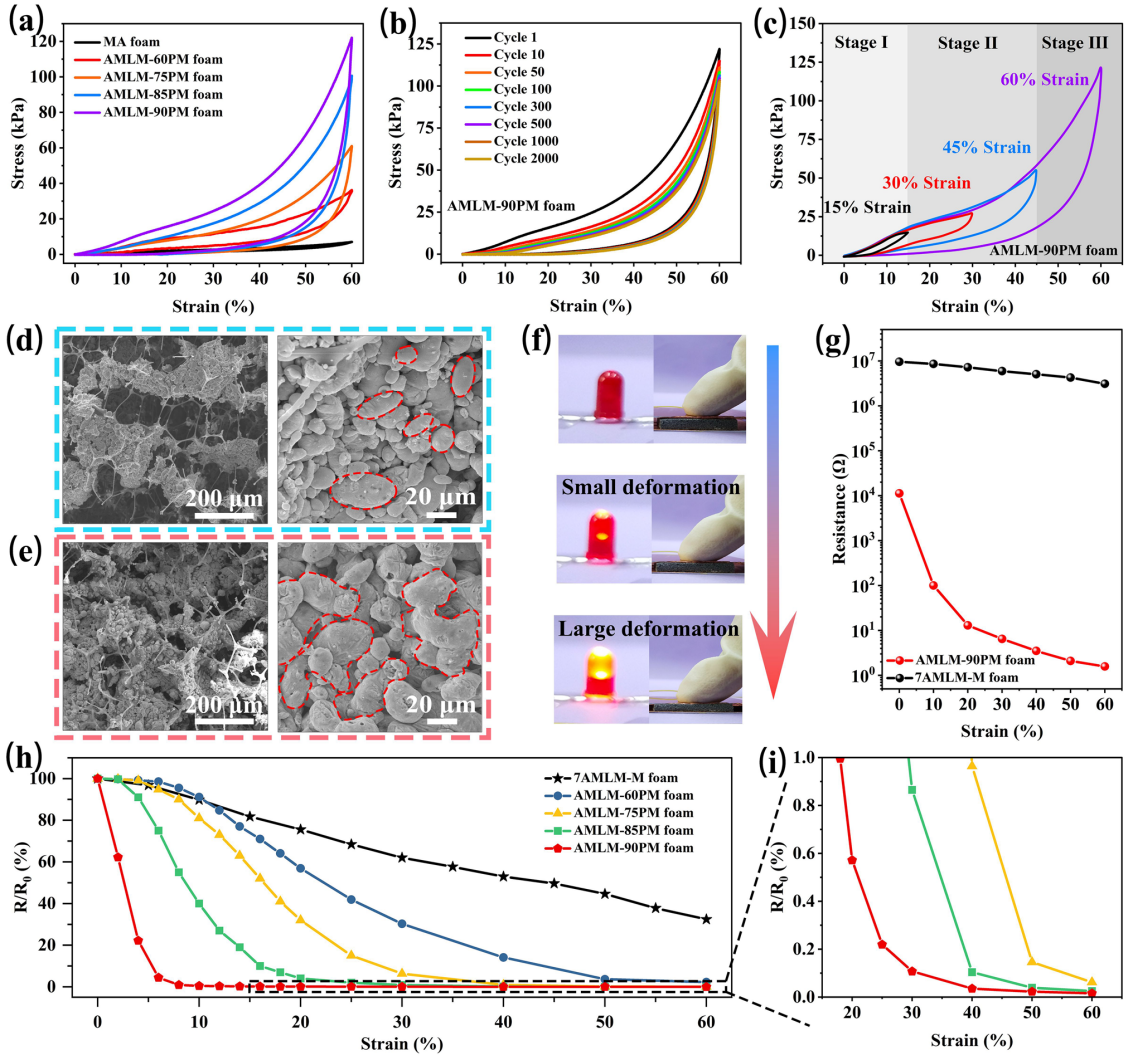
**a** Compressive stress–strain curves of MA foam and AMLM-*y*PM (*y* = 60, 75, 85, 90) foams during compression. **b** Stress–strain curves of AMLM-90PM foam during 2000 times of compression. **c** Stress–strain curves of AMLM-90PM foam under different compressive strains. **d-e** SEM images of AMLM-PM foam under different compressive strains of 0 and 60%. **f** Brightness change of LED bulb when compressing AMLM-90PM foam. **g** Variation of resistance of 7AMLM-M and AMLM-90PM foams with compressive strain. **h** Relative resistance (*R*/*R*_0_) of 7AMLM-M foam and AMLM-*y*PM (*y* = 60, 75, 85, 90) foams under different compressive strains. **i** Enlarged image of relative resistance of AMLM-*y*PM (*y* = 75, 85, 90) foams

Benefiting from the excellent elasticity of melamine foam, AMLM-PM foam can maintain good compression recoverability under appropriate AMLM adhesion amount. We first tuned the AMLM content to optimize the mechanical properties of composite foam (Table S2). By increasing the content of PMA films from 60, 75, 85, to 90 wt% (Fig. S15), more and more AMLM droplets can be loaded into the foam. The AMLM conductive films in foam gradually change from sparse distribution to densely interwoven distribution (Fig. S16). The corresponding samples are denoted as AMLM-60/75/85/90PM foams. Figure 3a shows the compressive stress–strain curves of these foams. MA foam shows a low compressive stress of 7.1 kPa under 60% compressive strain. With the increase of AMLM content, the compressive stress of AMLM-60PM, AMLM-75PM, AMLM-85PM, and AMLM-90PM foams increases to 36.1, 61.2, 100.6, and 122.4 kPa, respectively. Obviously, the loading of AMLM conductive film strengthens the foam skeleton, which significantly expands the pressure response range of AMLM-90PM foam. After unloading, the strain of all foams returned to zero, demonstrating their good elasticity. To demonstrate durability, Fig. 3b shows the stress–strain curves of AMLM-90PM foam during cyclic loading–unloading processes (at 60% strain). Even after 2000 cycles, the AMLM-90PM foam still maintains good recovery ability. The good durability of foam is essential for extending its service life in practical applications. It is worth noting that higher compressive strains of 65% and 70% adversely affect the mechanical stability of the foam (Fig. S17). However, if AMLM content further increases, the AMLM-95PM foam cannot return to its original state after compression (Figs. S18 and S19), indicating that excessive AMLM droplets will weaken the elasticity of the foam skeleton. Figure 3c shows the stress–strain curves of AMLM-90PM foam under different strain. There are three stages in the compression process, which is the characteristic of foam-like structure [36]. The initial linear stage at the strain below 15% demonstrates the elastic deformation of foam. In the middle strain range of 0–45%, the stress increases slowly due to the gradual compression of the macroporous structure of foam skeleton. At the high strain above 45%, the densification of foam results in a sharp increase of stress with strain. Figure 3d, e show the microstructure of uncompressed and compressed AMLM-90PM foam, respectively. At a large compressive strain of 60%, the shrinkage of foam voids causes a large number of AMLM droplets to agglomerate. The squeezed droplets transform from spherical/ellipsoidal shape to irregular shape. The good deformability of droplets can release local excessive stress, which is conducive to maintaining the integrity of foam skeleton. Therefore, foam can still maintain good compression recovery after loading a large number of AMLM droplets.

During compression, the deformation of foam will cause the change of resistance of foam with strain [37, 38]. Firstly, to visually observe this change, AMLM-90PM foam was connected to a circuit equipped with a LED bulb. The continuous compression of foam makes the bulb become brighter and brighter (Fig. 3f), indicating the decrease in the resistance of foam. Accurate resistance measurements show that as the compressive strain increases from 0 to 60%, the resistance of AMLM-90PM foam decreases from 11,257 to 1.6 Ω, i.e., almost four orders of magnitude (Fig. 3g). As shown in Fig. 3d, e, as the compressive strain increases, the separated AMLM conductive films gradually contact with each other to form a large area of conductive network, and meanwhile, the deformation of AMLM droplets further increases their contact area, which leads to a sharp drop in the resistance of foam. To further reveal the advantage of this inter-skeleton conductive films, we compared the resistance changes of AMLM-90PM foam (with conductive film) and the control samples *x*AMLM-M foam (with conductive skeleton) during compression, as shown in Figs. 3g and S20. As the compressive strain increases from 0 to 60%, the resistance of the representative 7AMLM-M foam only slightly decreases from 9.5 to 3.1 MΩ [39]. It suggests that the resistance change caused by “skeleton-skeleton” contact is much smaller than that caused by “film-film” contact. Therefore, the design of inter-skeleton conductive film can make foam have much higher resistance change at the same strain, which is conducive to significantly improving its functions.

Figure 3h, i compare the relative resistance *R*/*R*_0_ of AMLM-M foam and AMLM-*y*PM (*y* = 60, 75, 85, 90) foams. The *R*/*R*_0_ values of all the AMLM-*y*PM foams under 60% compressive strain are below 3%, far lower than that of AMLM-M foam (32%). But the decreasing rate of *R*/*R*_0_ value for various AMLM-*y*PM foam is obviously different. The *R*/*R*_0_ value of AMLM-90PM foam decreases fastest with strain, reaching below 1% at ~ 20% strain. This is because high content of AMLM conductive films easily form more contacts during compression, thus leading to a rapid change in resistance. In contrast, the *R*/*R*_0_ value of AMLM-60PM foam with the lowest AMLM content decreases slowly and reaches 3% at the maximum strain of 60%.

### Electromagnetic Interference Shielding Performance

Despite the progresses of compressible EMI shielding foams, the shielding performance (evaluated by total shielding effectiveness, i.e., SE_T_) of current foams will weaken during compression, which is a key problem to be solved urgently. SE_T_ includes the contributions from absorption (SE_A_) and reflection (SE_R_) [40–42]. According to Eqs. (7)–(9), the fundamental reasons for the decrease in SE_T_ are the reduction of SE_A_ caused by the decrease of foam thickness (${\text{SE}}_{{\text{A}}} \propto d$) [43–45]:

7 $$ {\text{SE}}_{{\text{T}}} {\text{ = SE}}_{{\text{R}}} {\text{ + SE}}_{{\text{A}}} $$

8 $$ {\text{SE}}_{{\text{R}}} = 20\log \left( {\frac{{\sqrt {\mu_{0} \sigma } }}{{4\sqrt {2\pi f\mu \varepsilon_{0} } }}} \right) $$

9 $$ {\text{SE}}_{{\text{A}}} = 8.68d\sqrt {\pi f\mu \sigma } $$

where *f* is frequency of EM waves; *μ* is the permeability of foam; *μ*_0_ and *ε*_0_ are the permeability and permittivity of free space, respectively, and *d* is the thickness of foam. In addition to changing thickness, compression also leads to an increase in conductivity, which can increase both SE_A_ and SE_R_. Therefore, the change of SE_T_ during compression depends on the competition between foam thickness and conductivity. However, due to the logarithm and square root relationships between SE_R_, SE_A_, and conductivity (${\text{SE}}_{{\text{R}}} \propto \log \left( {\sqrt \sigma } \right)$, ${\text{SE}}_{{\text{A}}} \propto d$), the increment of conductivity for current CPFs is insufficient during compression, which cannot compensate for the negative impact of thickness reduction. In fact, this theoretical analysis can also be confirmed by the decrease of EMI shielding performance of traditional *x*AMLM-M foams (with conductive skeletons) during compression, as shown in Fig. S21.

**Fig. 4:**
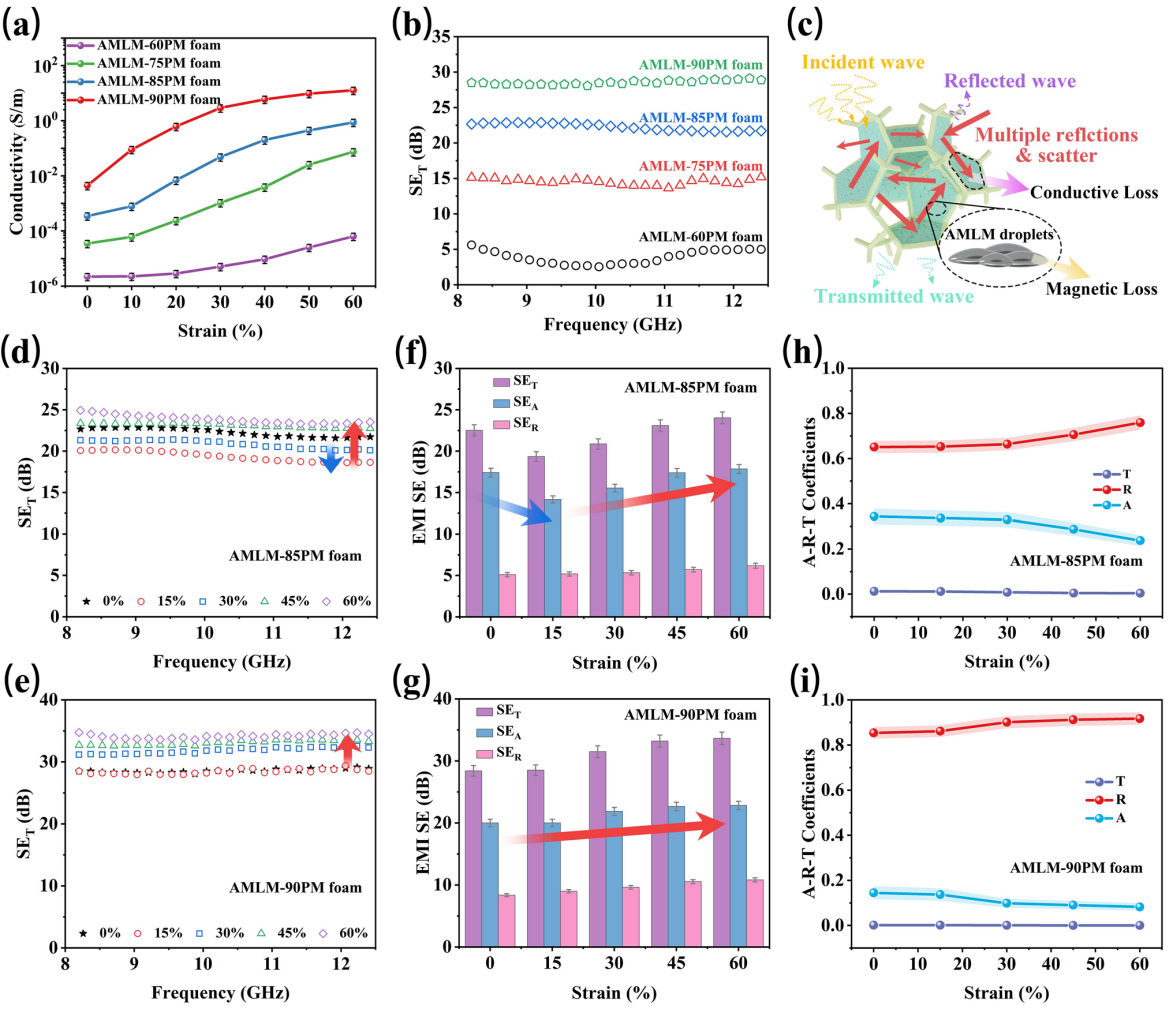
**a** Conductivity of AMLM-*y*PM (*y* = 60, 75, 85, 90) foams under different compressive strains. **b** EMI shielding curves of AMLM-*y*PM (*y* = 60, 75, 85, 90) foams. **c** Schematic illustration of EMI shielding mechanism of AMLM-*y*PM foam. EMI shielding curves of **d** AMLM-85PM and **e** AMLM-90PM foams during compression. Average SE_T_, SE_R_, and SE_A_ values of **f** AMLM-85PM and **g** AMLM-90PM foams under different compressive strains. Average *A*, *R,* and *T* values of **h** AMLM-85PM and **i** AMLM-90PM foams under different compressive strains

Benefiting from the design of inter-skeleton conductive film, the conductivity of AMLM-*y*PM foams can significantly increase during compression (Fig. 4a). The increase of AMLM contents leads to a more profound increase of conductivity of foam. As the compressive strain increases from 0 to 60%, the conductivity of AMLM-85PM foam increases from 3.4 × 10^–4^ to 0.6 S m^−1^, while the conductivity of AMLM-90PM foam astonishingly increases from 4.4 × 10^–3^ to 12 S m^−1^, an increase of four orders of magnitude. Such a large increase of conductivity may bring dawn to solve the above problem. Additionally, with the increase of compressive strain, the saturation magnetization and high-frequency permeability of AMLM-90PM foam increase (Fig. S22). These phenomena are typical characteristics of magneto-rheological foams [46, 47]. However, compared to the changes in conductivity and thickness, the change in permeability is quite small, demonstrating its minor effect on SE. The EMI shielding performances of the AMLM-*y*PM foams were then studied in detail. Figure 4b shows that with the increase of AMLM content, the shielding ability of the foam is enhanced, and the average SE_T_ values increase from ~ 3.6 to 28.5 dB, which is consistent with the change of conductivity. As illustrated in Fig. 4c, the shielding mechanism of AMLM-*y*PM foam derives from the direct reflection of EM wave on the foam surface, and the absorption of EM wave inside the foam mainly through conduction loss and magnetic loss [48–51]. In particular, compared with traditional CPFs with conductive skeleton, the inter-skeleton conductive films have the advantage of providing abundant interfaces for multiple reflections and scattering of EM wave, which promotes the attenuation of EM wave through increasing transmission paths.

Afterward, we tested the EMI shielding performances of AMLM-75PM, AMLM-85PM, and AMLM-90PM foams during compression. As shown in Fig. S23a, the SE_T_ value of AMLM-75PM continuously decreases as the strain increases to 30% and then gradually recovers to ~ 12.6 dB at 60% strain; however, it still remains lower than its original value. The SE_T_ value of AMLM-85PM initially decreases by 14% under a small strain of 15%, then increases with strain, and finally reaches ~ 24 dB under 60% strain, approaching the initial value (Fig. 4d). The SE_T_ value of the optimal AMLM-90PM remains unchanged under small strain and ultimately rises to 33.6 dB with increasing strain (Fig. 4e). To explain this phenomenon, we calculated the SE_R_ and SE_A_ values of the foams under different compressive strains. As shown in Figs. S23b and 4f, g, the decrease in SE_T_ of AMLM-75PM and AMLM-85PM foam under small strains originates from the considerable decrease in SE_A_, which can be attributed to the insufficient increase in conductivity due to the limited contact between AMLM films (Figs. S24). In this case, the weakening effect of thickness reduction on SE_A_ outweighs the enhancing effect of conductivity increase on SE_A_. In contrast, since more contacts between AMLM films easily form in compressed AMLM-90PM, the considerable increase of conductivity enables SE_A_ to stabilize under small strain and sharply increase under large strain (Figs. S24). Hence, the AMLM-90PM does not show a decline of SE_T_ during compression. We further calculated the power coefficients of absorptivity (*A*), reflectivity (*R*), and transmissivity (*T*) of AMLM-85PM and AMLM-90PM foams (Fig. 4h, i) [52–54]. Clearly, the *R* values are much higher than the *A* values, revealing the reflection-dominated shielding mechanism. The *T* values are as low as 10^−4^, suggesting that more than 99.99% of EMW is shielded by the foams. With the increase of compressive strain, *R* values gradually increase, which indicates a higher reflectivity of EM wave in compressed AMLM-85/90PM foams.

**Fig. 5:**
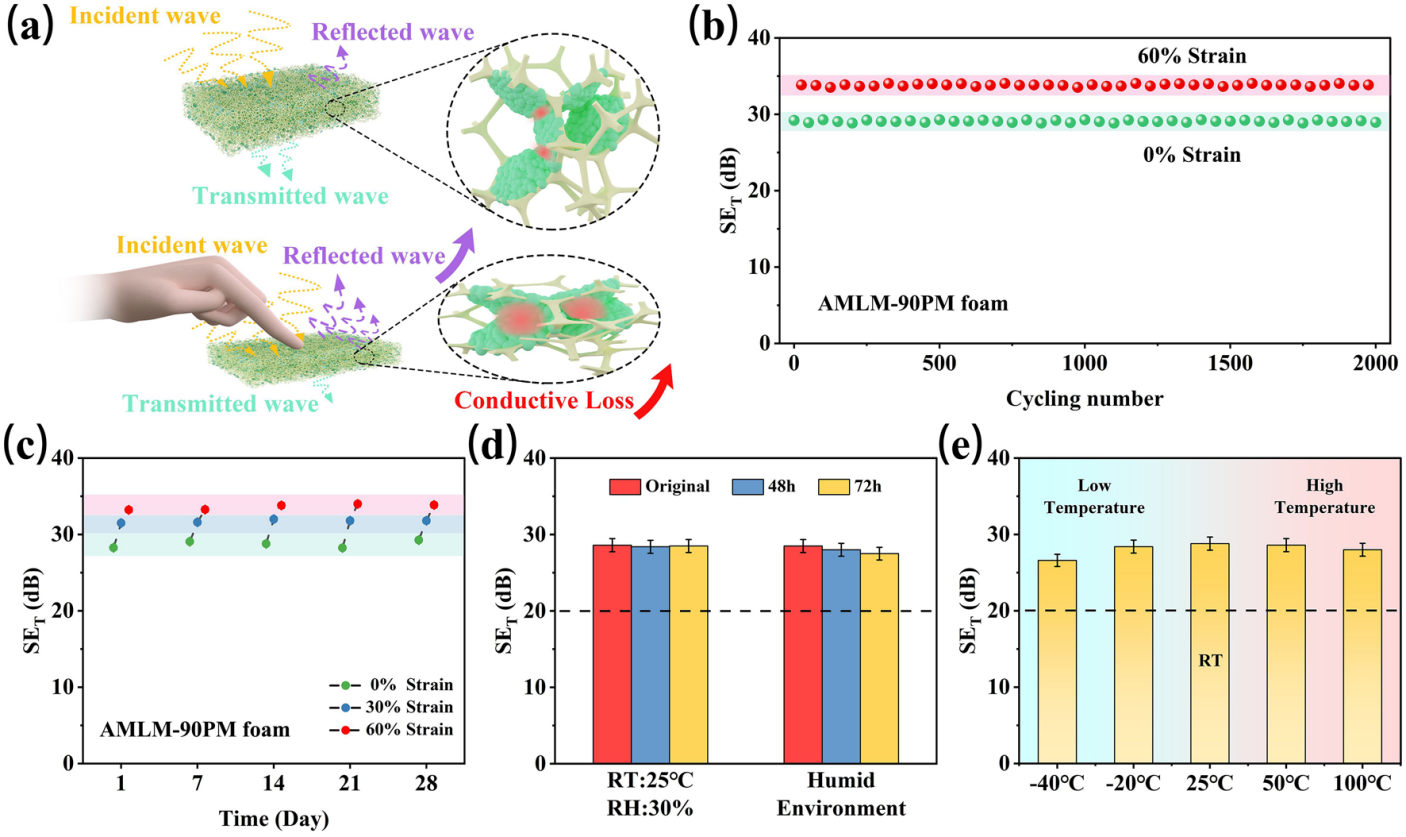
**a** Schematic illustration of shielding enhancement mechanism of compressed AMLM-PM foam. **b** Average SE_T_ values of AMLM-90PM foam under 0 and 60% strains during 2000 cycles of compression. **c** Changes in SE_T_ of AMLM-90PM foam with storage time under environmental conditions. **d** Average SE_T_ values of AMLM-90PM foam measured by storing the foam in different environments for 48 and 72 h (normal environment (RT = 25 °C, RH = 30%); humid environment (humidity: 80%)). **e** Average SE_T_ values of AMLM-90PM foam measured at different temperatures

According to the above analysis, the enhancement mechanism of shielding performance of compressed foam can be summarized in Fig. 5a. During compression, the decrease in foam thickness leads to a reduction of pore size, which decreases shielding performance by weakening the internal multiple reflection [55–57]. However, this negative effect can be offset by a sharp increase in conductivity. On the one hand, the sharp increase of conductivity under compression worsens impedance matching between foam and free space, causing more EM wave to be reflected on the foam surface [58–60]. On the other hand, the significant increase of conductivity can trigger stronger induced current in the compressed foam to dissipate the entered EM wave through enhanced conduction loss [61–63].

Stability, durability, and environmental adaptability are also necessary for the application of CPFs. We tested the EMI shielding performances of AMLM-90PM foam during 2000 compression-release cycles. Figure 5b shows the average SE_T_ values of the foam under uncompressed and compressed (60% strain) states. The SE_T_ values do not show significant fluctuations, indicating the durability of foam during repeated deformation. We stored AMLM-90PM foam at room temperature and tested the change of its shielding performance with storage time. As shown in Fig. 5c, the average SE_T_ of the AMLM-90PM foam remains stable for 28 days, and the average SE_T_ value can remain stable during the compression process. This indicates that AMLM droplets have high anti-oxidation ability due to the encapsulation of alginate layer. Furthermore, we placed the AMLM-90PM foam in different environments including humid environment (humidity: 80%) after 48 and 72 h, respectively. As shown in Fig. 5d, the average SE_T_ of AMLM-90PM foam does not show a significant decrease even after 72 h and stabilizes above 20 dB. Additionally, the EMI shielding performance of AMLM-90PM foam exhibits high stability over a wide temperature range of -40–100 °C (Fig. 5e). The excellent environmental adaptability enables the foam to be used in harsh environments and facilitates extending its service life. Table S3 summarizes the EMI shielding performances of CPF-based shielding materials. Compared with other shielding foams, AMLM-90PM foam not only shows impressive strain-adaptive shielding ability, but also shows the highest conductivity change under compression. This means that AMLM-90PM foam has superior advantages in integrating sensitive pressure sensing (discussed below).

### Pressure Sensing Performance

**Fig. 6:**
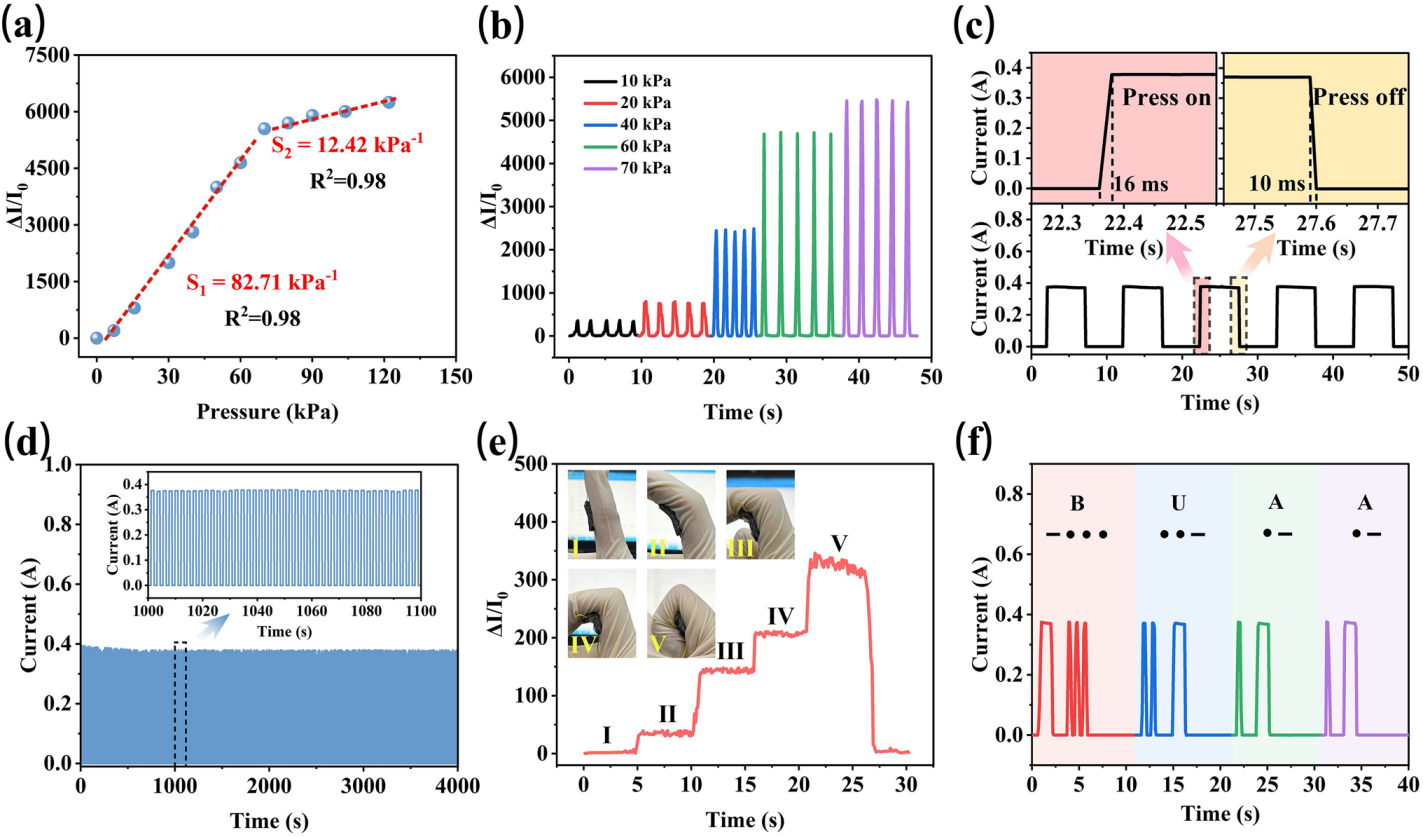
**a** Relative current change of AMLM-90PM foam under different pressures. **b** Δ*I*/*I*_0_ of the AMLM-90PM foam under cyclic loading at different pressure (10, 20, 40, 60, and 70 kPa). **c** Rapid current response of AMLM-90PM foam: response time of 16 ms and recovery time of 10 ms. **d** Current response of AMLM-90PM foam during 2000 compression cycles. **e** Relative current change of AMLM-90PM foam with different finger bending angles. **f** Current signals corresponding to BUAA Morse code, which is generated by pressing AMLM-90PM foam

The great change of resistance to strain also makes AMLM-90PM foam suitable for sensing external pressure. Combined with excellent strain-adaptive EMI shielding performance, the foam can be used as an anti-interference pressure sensor, which has the advantages of ensuring its sensing stability and high sensitivity. Relative current change Δ*I*/*I*_0_ × 100% of AMLM-90PM foam under different pressures is presented in Fig. 6a. As the pressure increases, the Δ*I*/*I*_0_ value of the foam monotonously increases. Sensitivity (*S* = (Δ*I*/*I*_0_)/Δ*P*) is an important indicator for evaluating the performance of sensor [64]. As shown in Figs. 6a and S25, the *S* value of AMLM-*y*PM foam increases with the increase of conductive component (*y* value). The AMLM-90PM foam has a very high *S* value of 82.71 kPa^−1^ in the pressure range of 0 ~ 70 kPa and a relatively high *S* value of 12.42 kPa^−1^ in the high-pressure range of 70 ~ 122.1 kPa. Table S4 compares the sensing performances of AMLM-90PM foam with other CPF-based pressure sensors. The high sensitivity and wide working pressure range of AMLM-90PM foam indicate its promising sensing applications. Figure 6b shows the different Δ*I*/*I*_0_ values of the foam under different pressures (10, 20, 40, 60, and 70 kPa). The Δ*I*/*I*_0_ value obviously increases with increasing pressure, returns to zero after releasing, and remains stable during continuous compression release. These results demonstrate the excellent stability and reversibility of the foam. Figure 6c shows the real-time current response of AMLM-90PM foam under compressive strain of 60%. Upon compression and release, the current increases and decreases sharply with a quite short response time of 16 and 10 ms, respectively, which indicates its rapid responsive ability. We further tested the current change of foam during 2000 cycles under 60% strain (Fig. 6d). The current maintains almost the same amplitude without significant changes, which demonstrates its high stability and durability.

Subsequently, AMLM-90PM foam was assembled into a simple wearable device to monitor human activities (Fig. S26a). This sensor can be adhered to different body parts. For example, it was adhered to the finger for real-time monitoring the bending of finger. As shown in Fig. 6e, a higher Δ*I*/*I*_0_ value indicates a larger bending degree of finger. At a fixed bending degree, the Δ*I*/*I*_0_ value keeps stable, suggesting the reliability of the monitor. Similarly, this device can also capture the movement of the elbow and knee (Fig. S26b, c). The sharp current peaks record the degree and rhythm of human movement. In addition, AMLM-90PM foam can be used to transmit Morse code, which is a key technique widely used in warfare and communication. As shown in Fig. 6f, the Morse code representing the English word “BUAA” is converted into current signals by controlling the pressing rhythm of AMLM-90PM foam. Additionally, in harsh environments including high temperature and humid environment, AMLM-90PM foam still maintains high sensitivity of 81.77 and 80.23 kPa^−1^, respectively, further indicating its excellent environmental adaptability (Fig. S27).

### Pressure-Tuned Joule Heating Performance

**Fig. 7:**
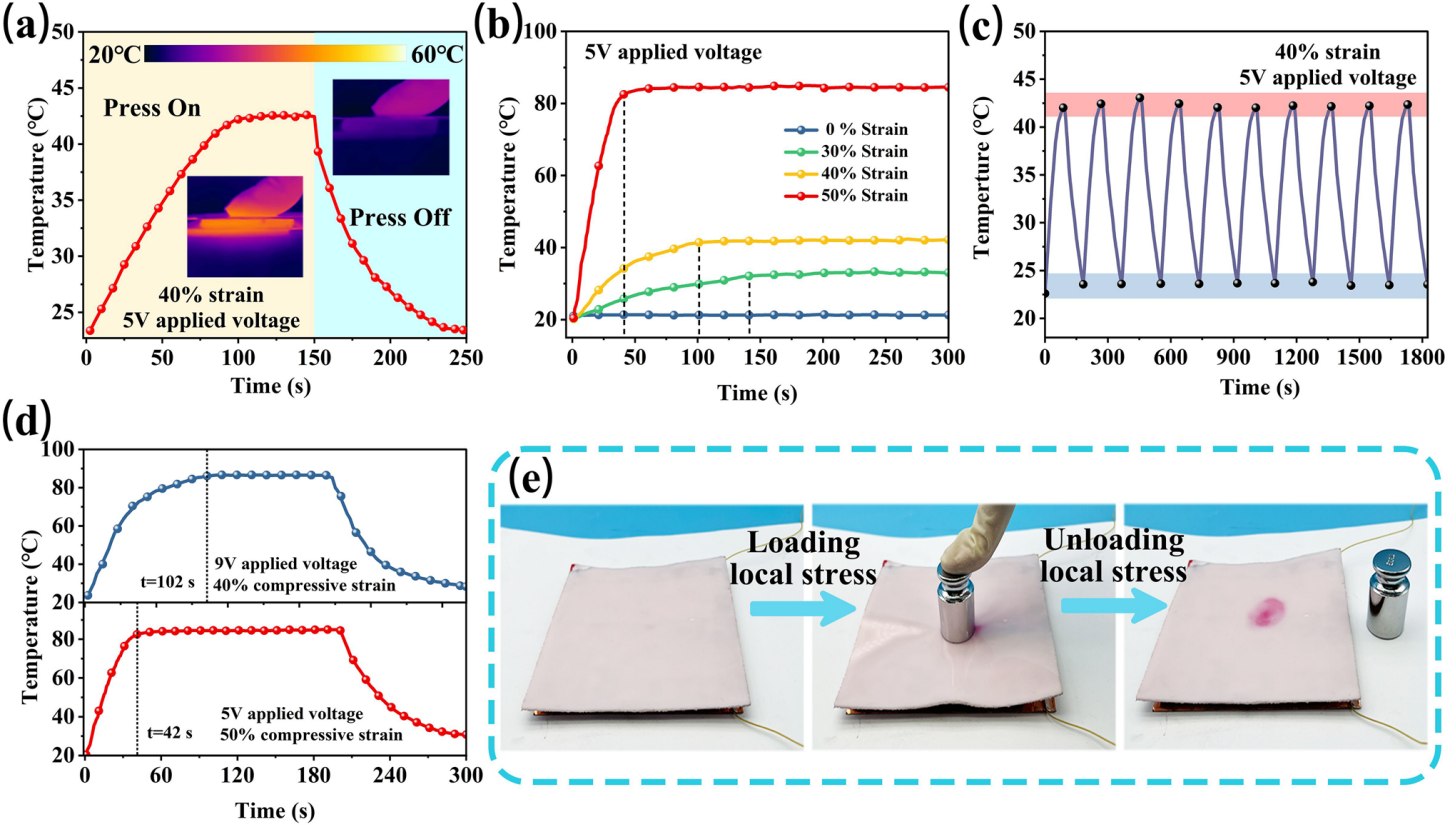
**a** Time-dependent surface temperature of AMLM-90PM foam under compressive strain of 40% (5 V applied voltage). **b** Temperature change of AMLM-90PM foam at 5 V under different compressive strains of 0, 30%, 40%, and 50%. **c** Temperature change of AMLM-90PM foam during cyclic compression release. **d** Fast and slow Joule heating methods to achieve a temperature of 87 °C. Upper curve: 40% strain, 9 V voltage; lower curve: 50% strain, 5 V voltage. **e** A scheme illustrating the structure of pressure indicating sensor, and photographs showing its working status

Smart thermal management achieved by adjusting temperature through mechanical operations is highly desirable for advanced wearable electronic devices. According to the Joule's law: *Q* = *U*^2^*t*/*R* (*Q* represents generated Joule, *U* is applied voltage, *t* is working time, and *R* represents resistance) [65, 66]. AMLM-90PM foam with compression-sensitive resistance is an idea candidate. As shown in Fig. 7a, under a fixed voltage of 5 V, once the AMLM-90PM foam is compressed, the surface temperature of the foam starts to increase and finally stabilizes at 42.5 °C within 102 s (40% strain). When the foam is released, its surface temperature gradually decreases to room temperature. The temperature of the foam shows a compression-regulated characteristic. Moreover, the saturation temperature and heating speed of AMLM-90PM foam can be controlled by changing the compressive strain. Figure 7b shows that when the applied voltage is 5 V, as the compressive strain increases from 30%, 40% to 50%, the saturation temperature of foam increases from 32, 42 to 84 °C within 142, 101, and 42 s, respectively. Generally, the temperature adjustment range required for wearable device is between room temperature and 40–50 °C. As shown in Fig. 7c, the temperature of AMLM-90PM foam can be sensitively switched between 23 and 42 °C by changing the compressive strain from 0 to 40% at a fixed voltage of 5 V. During repeated compression-release cycle, the temperature of foam does not show significant fluctuations, indicating its good durability. Figure S28 presents the change of surface temperature of AMLM-90PM foam with time (40% strain, 5 V). Even after 1800 s, the temperature of foam remains stable at ~ 42 °C, which suggesting its long-term stable Joule heating capability.

In addition, the surface temperature of AMLM-90PM foam under fixed compressive strain can be adjusted by changing the applied voltage (Fig. S29). More interestingly, the Joule heating behavior of AMLM-90PM foam can be synergistically controlled by strain and applied voltage to achieve fast and slow heating. As shown in Fig. 7d, under 40% compressive strain and 9 V applied voltage, the temperature of AMLM-90PM foam can slowly rise to 86 °C after 102 s. In comparison, under 50% compressive strain and 5 V applied voltage, the time for foam to reach the same temperature can be reduced to only 42 s. The dual regulation mode of voltage and stress enables AMLM-90PM foam to achieve different thermal management forms according to application requirements. In addition, this unique Joule heating characteristics of AMLM-90PM foam can be used to visually indicate applied force. As shown in Fig. 7e, the pressure indicator sensor, assembled by AMLM-90PM foam, electrode and thermochromic film, can accurately indicate the shape, area, and position of external force. When a local pressure (~ 50 kPa) was applied to the foam, the heat generated by the compressed AMLM-90PM foam led to the thermochromic film to change color. After removing the pressure, a clear red dot was left on the film, which accurately displays the pressure point. This function enables the pressure sensing of wearable devices to achieve visualization.

## Conclusions

We provide a new structural design strategy for CPFs to significantly improve its change amplitude of resistance with compressive strain. For the first time, conductive components are loaded on the pre-constructed inter-skeleton films in the polymer foam, which considerably increases the contact area and probability of conductive components under compression. As a result, four orders of magnitude of resistance decline were detected in the compressed foam, which is far superior to the tradition design. Meanwhile, the inter-skeleton conductive films exhibit advantages in improving mechanical properties, structural stability, and EM wave scattering. Correspondingly, this foam achieves strain-adaptive EMI shielding, which solves the problem of performance degradation of traditional CPFs, and integrates the functions of sensitive pressure sensing over a wide pressure range and compression-regulated Joule heating. In the future, it holds potential for wearable applications that can simultaneously monitor motion, provide thermal therapy, and prevent electromagnetic interference to ensure its high accuracy. This structural design strategy may inspire the preparation of more high-performance strain-responsive materials.

## Supplementary Information

Below is the link to the electronic supplementary material.

Supplementary file1 (DOC 14457 KB)

Supplementary file 2 (MP4 3625 kb)
