# Supplementary material for: Inter-Skeleton Conductive Routes Tuning Multifunctional Conductive Foam for Electromagnetic Interference Shielding, Sensing and Thermal Management
Source: Nanomicro Lett. 2024 Oct 28;17:52. doi: 10.1007/s40820-024-01540-z (PMC11513780; doi:10.1007/s40820-024-01540-z)
Supplement: Supplementary file 1 — Supplementary file1 (DOC 14457 KB) [file 40820_2024_1540_MOESM1_ESM.doc]

Supporting Information for

**Inter-Skeleton Conductive Routes Tuning Multifunctional Conductive Foam for Electromagnetic Interference Shielding, Sensing and Thermal Management**

Xufeng Li^1^, Chunyan Chen^1^, Zhenyang Li^1^, Peng Yi^1^, Haihan Zou^1^, Gao Deng^1^, Ming Fang^1^, Junzhe He^4^, Xin Sun^4^, Ronghai Yu^1^, *, Jianglan Shui^1, 3^, *, Caofeng Pan ^2^, *, and Xiaofang Liu^1^, *

^1^ School of Materials Science and Engineering, Beihang University, Beijing, 100191, P. R. China

^2^ Institute of Atomic Manufacturing, Beihang University, Beijing, 100191, P. R. China

^3^ Tianmushan Laboratory, Xixi Octagon City, Yuhang District, Hangzhou, 310023, P. R. China

^4^ Science and Technology on Electromagnetic Scattering Laboratory, Beijing Institute of Environmental Features, Beijing 100854, P. R. China

*Corresponding authors. E-mail: [rhyu@buaa.edu.cn](mailto:rhyu@buaa.edu.cn) (Ronghai Yu); [pancaofeng@buaa.edu.cn](mailto:pancaofeng@buaa.edu.cn) (Caofeng Pan); [shuijianglan@buaa.edu.cn](mailto:shuijianglan@buaa.edu.cn) (Jianglan Shui); [liuxf05@buaa.edu.cn](mailto:liuxf05@buaa.edu.cn) (Xiaofang Liu)

**Supplementary Figures and Tables**





**Fig. S1** SEM images of **a** PM foam and **b** AMLM-PM foam





**Fig. S2** **a-b** SEM images of PM foam in initial state. **c-d** SEM images of PM foam under compression. **e-f** SEM images of PM foam after releasing the pressure





**Fig. S3** XRD patterns of LM, ALM (alginate-modified LM), MLM (magnetic LM) and AMLM (alginate-modified magnetic LM) droplets

For MLM and AMLM, in addition to the diffraction peaks from galinstan and iron, no diffraction peaks from Fe-based compound were identified, indicating physical mixing of Fe particles and LM.





**Fig. S4** **a** Photos showing the difference of LM and MLM. **b** SEM image of ALM droplets after vigorous stirring. **c** SEM image of AMLM droplets after vigorous stirring. **d** SEM image and EDS mapping results of AMLM droplets





**Fig. S5** **a, b** Photos of ALM-PM foam in uncompressed and compressed states, respectively. **c, d** Photos of AMLM-PM foam in uncompressed and compressed states, respectively. **e** Photos showing that ALM droplets was squeezed out during compression. **f** Photo showing that AMLM droplets was not squeezed out during compression





**Fig. S6** Magnetization curve of AMLM droplets

**

**

**Fig. S7** **a** Illustration of coating a layer of sodium alginate on MLM droplet. **b** TEM image of an AMLM droplet





**Fig. S8** High-resolution Ga 2p _3/2_ of AMLM droplets

According to the Ga 2p_3/2_ spectrum in Fig. S8, gallium on the surface of AMLM droplets exists in Ga^3+^ states, suggesting the formation of gallium alginate gel.

The formation of gallium alginate shell on MLM droplets can be explained as follows [S1]. Firstly, metallic Ga is easily oxidized in air, forming a layer of Ga_2_O_3_ on the surface of LM [S2-S4]. When mechanical shear forces damage large blocks of MLM, the specific surface area of MLM greatly increases, and the newly exposed Ga metal reacts chemically with H_2_O and O_2_ to generate GaOOH. The reaction mechanism is shown in Eq. (S1):

$\text{4Ga + 2H}_{2}\text{O + 3O}_{2}\text{ = 4GaOOH}$ (S1)

Subsequently, the corresponding ionization reaction occurs to release gallium ions as shown in Eq. (S2):

$\text{GaOOH + H}_{2}\text{O }\leftrightarrow\text{ Ga}^{3+}\text{ + 3OH}^{-}$ (S2)

Alginate is composed of 1−4-linked α-Lguluronic (G) and β-D-mannuronic (M) acid residues. Gallium ions generated by ionization reaction will replace sodium ions on the carboxyl group of sodium alginate, connecting each alginate chain with many other chains, thus forming a gallium alginate gel network. Under continuous mechanical shear, the micro gel generated by gallium ion cross-linking is wrapped on the newly generated MLM micro drops, forming a core-shell structure.

**Optimizing the weight ratio of AMLM droplets to PMA films**

Taking AMLM-85PM foam (the weight ratio of PMA in PM foam is 85%) as an example, different amounts of AMLM droplets were loaded on the PMA films (Table S1). The weight ratio (*Q*) of AMLM droplets to PMA films was adjusted from 3 to 9. As the *Q* value increases, more and more AMLM droplets adhere to the PMA films (Fig. S9). When *Q* = 7, AMLM droplets can completely cover the PMA films. However, when *Q* = 9, AMLM droplets show severe agglomeration, indicating excessive loading.

As shown in Figs. S10a and S10b, the increase in the number of AMLM droplets on the PMA films leads to a significant increase in the resistance change during the compression process, which is related to the formation of more conductive contact points due to the increasing number of AMLM droplets adhering to the PMA film. The conductivity change of the foam during the compression process is closely related to the change of EMI shielding performance. As shown in Fig. S10c, the AMLM-85PM foam with less liquid metal addition on PMA films will have a significant reduction in SE_T_ values during the compression process due to insufficient electrical conductivity compensation. The increasing amount of AMLM droplets on PMA films leads to a gradually apparent compensatory effect of conductivity, thus weakening the reduction of SE_T_ values during the compression process.

Figure S11 shows the photos of compressing AMLM-85PM foam (*Q* = 7) and AMLM-85PM foam (*Q* = 9). When AMLM-85PM foam (*Q* = 9) was compressed, excessive AMLM droplets were easily squeezed out. However, for the AMLM-85PM foam (*Q* = 7), AMLM droplets were not squeezed out during compression. Therefore, *Q* = 7 is considered the optimal weight ratio of AMLM droplets to PM foam.

**

**

**Fig. S9** SEM images of AMLM-85PM foams with different *Q* values: **a** *Q* = 3, **b** *Q* = 5, **c** *Q* = 7, **d** *Q* = 9





**Fig. S10** **a** Resistance and **b** conductivity changes of AMLM-85PM foams with different *Q* values during compression. **c** Average SE_T_ values of AMLM-85PM foams with different *Q* values during compression

**

**

**Fig. S11** Photos showing the compression of AMLM-85PM foams with different *Q* values: **a** *Q* = 7, **b** *Q* = 9

**

**

**Fig. S12** XPS spectra of AMLM droplets, PM foam and AMLM-PM foam

**

**

**Fig S13** The relationship between the contact angle and the surface tension

The relationship between the contact angle and the surface tension can be described by the following two equations:

(S3)

(S4)

where *σ*_SG_, *σ*_SL_, and *σ*_LG_ are the surface tensions of solid–gas interface, solid–liquid interface, and liquid–gas interface, *θ* is the contact angle. Equation (S4) indicates the relationship between the adhesion work and the surface tension. Combining Eqs. (S3) and (S4) together, adhesion work (*W*_SL_) can be described by Equation (S5), which is called the Young-Dupre Equation:

(S5)

According to the previous study, the *σ*_LG_ of EGaIn is 624 mN/m [S5].

**

**

**Fig. S14** Physical images of AMLM-PM foam during compression

**

**

**Fig. S15** SEM images of **a, b** 60PM foam; **c, d** 75PM foam; **e, f** 85PM foam; **g, h** 90PM foam

**

**

**Fig. S16** SEM images of **a** AMLM-60PM foam; **b** AMLM-75PM foam; **c** AMLM-85PM foam; **d** AMLM-90PM foam





**Fig. S17** **a** Height retention and **b** Stress retention of AMLM-90PM over 2000 compression cycles with a compressive strain under different compression strains (55%, 60%, 65%, 70%)

The results show that when the compressive strain reaches 60%, the height and stress retention rates of foam are ~90%, indicating that AMLM-90PM foam has good elasticity. However, if the compressive strain exceeds 60%, both the height and stress retention significantly decrease. This indicates that higher strains adversely affect the mechanical stability of the foam. Therefore, it is appropriate to select a compressive strain of 60% (corresponding to a stress of ~120 kPa).





**Fig. S18** Cyclic stress-strain curves of AMLM-95PM foam at compressive strain of 60%

After releasing stress, the residual strain of AMLM-95PM foam is 28%, much larger than that of AMLM-90PM (2%). The results demonstrate the severe plastic deformation of the foam skeleton due to the excessive AMLM droplets.

**

**

**Fig. S19 a-c** Photos showing the compression and recovery of AMLM-90PM foam. **d-f** Photos showing the compression and recovery of AMLM-95PM foam





**Fig. S20** Relative resistance of *x*AMLM-M foams under different compressive strains

We prepared three *x*AMLM-M foams (*x* = 7, 14, 28) as control samples by loading different amount of AMLM droplets on the skeletons of melamine foams without PMA films. *x* represents the weight ratio of AMLM droplets to M foam. *x*AMLM-M foams have traditional conductive skeleton structure (as shown in Fig. 2b). It can be seen from Fig. S20 that 7AMLM-M foam shows the largest resistance reduction among the three samples. Therefore, 7AMLM-M foam is further compared with AMLM-*y*PM foams in Fig. 3g, h.





**Fig. S21 a** EMI shielding curves of *x*AMLM-M (*x* = 7, 14, 28) foams. **b** Schematic illustration of EMI shielding mechanism of AMLM-M foam. **c** Average SE_T_ values of *x*AMLM-M (*x* = 7, 14, 28) foams under 0% and 60% compressive strains. **d** Change of conductivity of *x*AMLM-M (*x* = 7, 14, 28) foams with compressive strains

As shown in Fig. S21a, the EMI shielding performance of *x*AMLM-M foam increases with increasing AMLM amount. Fig. S21c shows that the EMI shielding performance of all the *x*AMLM-M foams decreases under 60% compressive strain, which is due to the insufficient increase of conductivity of AMLM-M foam under compression (Fig. S21d).





**Fig. S22 a** Magnetization curves of AMLM-90PM foam at 0%, 30% and 60% compressive strains. **b** High-frequency permeability of AMLM-90PM foam at 0%, 30% and 60% compressive strains





**Fig. S23** **a** EMI shielding curves of AMLM-75PM foams during compression. **b** Average SE_T_, SE_R_ and SE_A_ values of AMLM-75PM foam under different compressive strains. **c** Variation of EMI SE values of AMLM-75PM, AMLM-85PM and AMLM-90PM foams under small strain (15% strain)





**Fig. S24** The changes in conductivity and EMI SE of **a** AMLM-75PM; **b** AMLM-85PM and **c** AMLM-90PM foams during compression

**

**

**Fig. S25** Relative current change under different pressures: **a** AMLM-60PM foam; **b** AMLM-75PM foam and **c** AMLM-85PM foam

**

**

**Fig. S26 a** Schematic illustration of wearable device based on AMLM-90PM foam. **b, c** Relative current changes of AMLM-90PM foam when repeatedly bending elbow and knee

**

**

**Fig. S27** Relative current change of AMLM-90PM foam under different pressures, which were tested in different harsh environments (normal environment (RT = 25 ^o^C, RH = 30%); high temperature (100 ^o^C) and humid environment (humidity: 80%))





**Fig. S28** Temperature change curve and infrared thermal images of AMLM-90PM foam during long-term heating (40% strain, 5 V)





**Fig. S29** Change of surface temperature with time for AMLM-90PM foam under different applied voltages (at 40% strain)

When the compression strain was stabilized at 40%, the saturation temperature (*T*_S_) of the AMLM-90PMA foam rose from 25 ^o^C to 87 ^o^C as the applied voltage was increased from 1V to 9V (Fig. S29). As the applied voltage of the AMLM-90PMA foam is incrementally raised, more electrons collide with the atomic lattice during the movement to generate more heat energy, resulting in a higher Joule heating temperature.

**Table S1 Weight ratio of AMLM droplets to PMA films in AMLM-85PM foams**

| Sample *^a^* | *M*_(PMA)_ (g) *^b^* | *M*_(AMLM)_ (g) *^c^* | weight ratio (*Q*) *^d^* |
| --- | --- | --- | --- |
| AMLM-85PM foam | 0.185 | 0.555 | 3 |
|  | 0.193 | 0.965 | 5 |
|  | 0.192 | 1.344 | 7 |
|  | 0.190 | 1.710 | 9 |

*^a^* AMLM-85PMfoam were cut into 1.5 × 3.0 × 0.5 cm^3^; *^b^* weight of PMA film; *^c^* weight of AMLM droplets; *^d^* weight ratio (*Q*) of AMLM droplets to PMA film

(S6)

**Table S2 Composition of AMLM-*y*PM foams**

| Sample *^a^* | weight ratio (*Q*) *^b^* | *M*_(PMA)_ (g) *^c^* | *M*_(AMLM)_ (g) *^d^* |
| --- | --- | --- | --- |
| AMLM-60PM foam | 7 | 0.065 | 0.455 |
| AMLM-75PM foam | 7 | 0.104 | 0.728 |
| AMLM-85PM foam | 7 | 0.182 | 1.274 |
| AMLM-90PM foam | 7 | 0.260 | 1.820 |

*^a^* All foams were cut into 1.5 × 3.0 × 0.5 cm^3^; *^b^* weight ratio (*Q*) of AMLM droplets to PMA film; *^c^* weight of PMA film; *^d^* weight of AMLM droplets

**Table S3** Comparison of EMI shielding performance of CPFs

| SE change under compression | Material | Strain | Change of resistance/conductivity | Refs. |
| --- | --- | --- | --- | --- |
| Weakened | EVA@ PPy@AgNPs foam | 0 - 50% |  | [S6] |
|  | PU/graphene foam | 0 - 75% |  | [S7] |
|  | MF@MA/PEG foam | 0 - 50% |  | [S8] |
|  | TPI-M/CF foam | 0 - 60% |  | [S9] |
|  | cGF/ABS foam | 0 - 75% |  | [S10] |
|  | PDMS/TSM/CNT foam | 0 - 70% |  | [S11] |
|  | SWNT/GA-chitosan polymer foam | 0 - 75% |  | [S12] |
|  | PU/CNT foam | 0 - 80% |  | [S13] |
|  | WF/CNT foam | 0 - 70% | ΔR/R_0_ ≈ 73% | [S14] |
|  | CNT/PU@PU foam | 0 - 75% | Δσ/σ_0_ ≈ 140 times | [S15] |
| Enhanced | PDMS/LM foam | 0 - 60% | Δσ/σ_0_ ≈ 14 times | [S16] |
|  | Wood/XC-72 sponge | 0 - 75% | Δσ/σ_0_ ≈ 43 times | [S17] |
|  | CrO_2_@G-LM asymmetric foam | 0 - 90% | Δσ/σ_0_ ≈ 8 times | [S18] |
|  | AMLM-90PM foam | 0 - 60% | Δσ/σ_0_ ≈ **2700 times** | This work |

**Table S4** Comparison of sensitivity and working pressure range of AMLM-90PM foam with other CPF-based pressure sensors

| Material | Method | Sensitivity (kPa^-1^) | Range (kPa) | Refs. |
| --- | --- | --- | --- | --- |
| TPU@ rGO-Ag NPs@PU sponge | Immersing | 152.97 | 0 ~ 40 | [S19] |
| rGO/polyaniline sponge | Dipping | 0.152 | 0 ~ 27.39 | [S20] |
| MS-PANI-A sponge | Cryopolymerization | 0.54 | 0 ~ 100 | [S21] |
| CNT/PU@PU sponge | Dipping | 16.17 | 0 ~ 300 | [S15] |
| DPyCF@SR | Carbonization + structural assembly | 24.6 | 0 ~ 14000 | [S22] |
| CNTs/PDMS foam | Immersing | 290.45 | 0 ~ 270 | [S23] |
| CB@PU sponges | Dipping | 0.068 | 0 ~ 17 | [S24] |
| MWNT− rGO@PU sponge | Dipping | 0.088 | 0 ~60 | [S25] |
| Au@PU sponge | Ion sputtering | 0.122 | 0 ~14.2 | [S26] |
| NOC PPy/PDA/PU foam | Dipping + structural design | 0.8251 | 0 ~ 25 | [S27] |
| Ag sponge | Chemical growth | 0.013 | 0 ~85 | [S28] |
| p-PU/CNF@CB foam | Plasma method | 0.35 | 0 ~30 | [S29] |
| AMLM-90PM foam | Dipping | 82.71 | 0 ~ 122.1 | This work |

**Supplementary References**

1. P. Wu, J. Fu, Y. Xu, Y. He, Liquid metal microgels for three-dimensional printing of smart electronic clothes. ACS Appl. Mater. Interfaces **14**, 13458-13467 (2022). <https://doi.org/10.1021/acsami.1c22975>
2. H. Song, T. Kim, S. Kang, H. Jin, K. Lee, et al. Ga‐based liquid metal micro/nanoparticles: Recent advances and applications. Small **16**, 1903391 (2019). <https://doi.org/10.1002/smll.201903391>
3. S. Zhao, J. Zhang, L. Fu. Liquid metals: A novel possibility of fabricating 2D metal oxides. Adv Mater. **33**, e2005544 (2021). <https://doi.org/10.1002/adma.202005544>
4. J. H. Fu, T. Y. Liu, Y. Cui, J. Liu, Interfacial engineering of room temperature liquid metals. Adv. Mater. Interfaces **8**, 2001936 (2021). <https://doi.org/10.1002/admi.202001936>
5. R. Guo, J. Tang, S. Dong, J. Lin, H. Wang, et al. One‐step liquid metal transfer printing: Toward fabrication of flexible electronics on wide range of substrates. Adv. Mater. Technol. **3**, 1800265 (2018). <https://doi.org/10.1002/admt.201800265>
6. J. Chen, W.-j. Jiang, Z. Zeng, D.-x. Sun, X.-d. Qi, et al. Multifunctional shape memory foam composites integrated with tunable electromagnetic interference shielding and sensing. Chem Eng J. **466**, 143373 (2023). <https://doi.org/10.1016/j.cej.2023.143373>
7. B. Shen, Y. Li, W. Zhai, W. Zheng, Compressible graphene-coated polymer foams with ultralow density for adjustable electromagnetic interference (EMI) shielding. ACS Appl. Mater. Interfaces **8**, 8050-8057 (2016). <https://doi.org/10.1021/acsami.5b11715>
8. Y.-j. He, Y.-w. Shao, Y.-y. Xiao, J.-h. Yang, X.-d. Qi, et al. Multifunctional phase change composites based on elastic MXene/silver nanowire sponges for excellent thermal/solar/electric energy storage, shape memory, and adjustable electromagnetic interference shielding functions. ACS Appl. Mater. Interfaces **14**, 6057-6070 (2022). <https://doi.org/10.1021/acsami.1c23303>
9. X. Jia, B. Shen, L. Zhang, W. Zheng, Construction of shape-memory carbon foam composites for adjustable EMI shielding under self-fixable mechanical deformation. Chem Eng J. **405**, 126927 (2021). <https://doi.org/10.1016/j.cej.2020.126927>
10. L. Wang, Y. Wu, Y. Wang, H. Li, N. Jiang, et al. Laterally compressed graphene foam/acrylonitrile butadiene styrene composites for electromagnetic interference shielding. Composites, Part A **133**, 105887 (2020). <https://doi.org/10.1016/j.compositesa.2020.105887>
11. J.-H. Cai, X.-H. Tang, X.-D. Chen, M. Wang. Temperature and strain-induced tunable electromagnetic interference shielding in polydimethylsiloxane/multi-walled carbon nanotube composites with temperature-sensitive microspheres. Composites, Part A **140**, 106188 (2021). <https://doi.org/10.1016/j.compositesa.2020.106188>
12. S. Zhu, Q. Zhou, M. Wang, J. Dale, Z. Qiang, et al. Modulating electromagnetic interference shielding performance of ultra-lightweight composite foams through shape memory function. Composites, Part B **204**, 108497 (2021). <https://doi.org/10.1016/j.compositesb.2020.108497>
13. G. Wang, D. Yi, X. Jia, J. Chen, B. Shen, et al. Structural design of compressible shape-memory foams for smart self-fixable electromagnetic shielding with reduced reflection. Mater. Today Phys. **22**, 100612 (2022). <https://doi.org/10.1016/j.mtphys.2022.100612>
14. Y. Chen, Y. Liu, Y. Li, H. Qi. Highly sensitive, flexible, stable, and hydrophobic biofoam based on wheat flour for multifunctional sensor and adjustable EMI shielding applications. ACS Appl. Mater. Interfaces **13**, 30020-30029 (2021). <https://doi.org/10.1021/acsami.1c05803>
15. X. Zhang, K. Wu, G. Zhao, H. Deng, Q. Fu. The preparation of high performance multi-functional porous sponge through a biomimic coating strategy based on polyurethane dendritic colloids. Chem. Eng. J. **438**, 135659 (2022). <https://doi.org/10.1016/j.cej.2022.135659>
16. B. Yao, X. Xu, H. Li, Z. Han, J. Hao, et al. Soft liquid-metal/elastomer foam with compression-adjustable thermal conductivity and electromagnetic interference shielding. Chem. Eng. J. **410**, 128288 (2021). <https://doi.org/10.1016/j.cej.2020.128288>
17. X. Liu, Y. Li, X. Sun, W. Tang, G. Deng, et al. Off/on switchable smart electromagnetic interference shielding aerogel. Matter **4**, 1735-1747 (2021). <https://doi.org/10.1016/j.matt.2021.02.022>
18. C. Yuan, X. Li, M. Huang, F. Li, Z. Zhang, et al. Multifunctional asymmetric foam with compression-enhanced EMI shielding effectiveness exhibits real-time tunability of reflection/absorption ratio and pressure sensing. Chem. Eng. J. **489**, 151359 (2024). <https://doi.org/10.1016/j.cej.2024.151359>
19. M. Cao, M. Leng, W. Pan, Y. Wang, S. Tan, et al. 3D wearable piezoresistive sensor with waterproof and antibacterial activity for multimodal smart sensing. Nano Energy **112**, 108492 (2023). <https://doi.org/10.1016/j.nanoen.2023.108492>
20. G. Ge, Y. Cai, Q. Dong, Y. Zhang, J. Shao, et al. A flexible pressure sensor based on rgo/polyaniline wrapped sponge with tunable sensitivity for human motion detection. Nanoscale **10**, 10033-10040 (2018). <https://doi.org/10.1039/c8nr02813c>
21. L. Li, X. Bao, J. Meng, C. Zhang, T. Liu, Sponge-hosting polyaniline array microstructures for piezoresistive sensors with a wide detection range and high sensitivity. ACS Appl. Mater. Interfaces **14**, 30228-30235 (2022). <https://doi.org/10.1021/acsami.2c07404>
22. R. Chen, T. Luo, J. Wang, R. Wang, C. Zhang, et al. Nonlinearity synergy: An elegant strategy for realizing high-sensitivity and wide-linear-range pressure sensing. Nat. Commun. **14**, 6641 (2023). <https://doi.org/10.1038/s41467-023-42361-9>
23. X. Lei, L. Ma, Y. Li, Y. Cheng, G. J. Cheng, et al. Highly sensitive and wide-range flexible pressure sensor based on carbon nanotubes-coated polydimethylsiloxane foam. Mater. Lett. **308**, 131151 (2022). <https://doi.org/10.1016/j.matlet.2021.131151>
24. X. Wu, Y. Han, X. Zhang, Z. Zhou, C. Lu. Large‐area compliant, low‐cost, and versatile pressure‐sensing platform based on microcrack‐designed carbon black@polyurethane sponge for human–machine interfacing. Adv. Funct. Mater. **26**, 6246-6256 (2016). <https://doi.org/10.1002/adfm.201601995>
25. A. Tewari, S. Gandla, S. Bohm, C. R. McNeill, D. Gupta, Highly exfoliated MWNT-rGO ink-wrapped polyurethane foam for piezoresistive pressure sensor applications. ACS Appl. Mater. Interfaces **10**, 5185-5195 (2018). <https://doi.org/10.1021/acsami.7b15252>
26. Y.-h. Wu, H.-z. Liu, S. Chen, X.-c. Dong, P.-p. Wang, et al. Channel crack-designed gold@PU sponge for highly elastic piezoresistive sensor with excellent detectability. ACS Appl. Mater. Interfaces **9**, 20098-20105 (2017). <https://doi.org/10.1021/acsami.7b04605>
27. X. Ren, Q. Tian, X. Zhu, H.-Y. Mi, X. Jing, et al. Multi-scale closure piezoresistive sensor with high sensitivity derived from polyurethane foam and polypyrrole nanofibers. Chem. Eng. J. **474**, 145926 (2023). <https://doi.org/10.1016/j.cej.2023.145926>
28. L. Wang, D. Wang, Z. Wu, J. Luo, X. Huang, et al. Self-derived superhydrophobic and multifunctional polymer sponge composite with excellent joule heating and photothermal performance for strain/pressure sensors. ACS Appl. Mater. Interfaces **12**, 13316-13326 (2020). <https://doi.org/10.1021/acsami.0c00150>
29. S. Xu, X. Li, G. Sui, R. Du, Q. Zhang, et al. Plasma modification of PU foam for piezoresistive sensor with high sensitivity, mechanical properties and long-term stability. Chem. Eng. J. **381**, 122666 (2020). <https://doi.org/10.1016/j.cej.2019.122666>
